# Creep Behavior of CLT Beams with Finite Thickness Layers of Flexible Adhesives

**DOI:** 10.3390/ma16124484

**Published:** 2023-06-20

**Authors:** Klaudia Śliwa-Wieczorek, Paweł Szeptyński, Tomasz Kozik, Martino Gubert

**Affiliations:** 1 Division of Bridge, Metal and Timber Structures, Faculty of Civil Engineering, Cracow University of Technology, 31-155 Cracow, Poland; 2 Division of Structural Mechanics and Material Mechanics, Faculty of Civil Engineering, Cracow University of Technology, 31-155 Cracow, Poland; pawel.szeptynski@pk.edu.pl; 3 PalettenWerk Kozik Sp. z o. o., 34-240 Jordanów, Poland; 4 Eurac Research, 39100 Bolzano, Italy; martino.gubert@eurac.edu

**Keywords:** CLT beams, wood, flexible adhesives, creep, viscoelasticity

## Abstract

Creep behavior of Cross-Laminated-Timber (CLT) beams with a finite-thickness layer of flexible adhesives is investigated. Creep tests were carried out for all component materials as well as for the composite structure itself. Three-point bending creep tests were performed for spruce planks and for CLT beams, and uniaxial compression creep tests were performed for two flexible polyurethane adhesives: Sika^®^ PS and Sika^®^ PMM. All materials are characterized with the use of the three-element Generalized Maxwell Model. The results of creep tests for component materials were used in elaboration of the Finite Element (FE) model. The problem of linear theory of viscoelasticity was solved numerically with the use of the Abaqus software. Obtained results of Finite Element Analysis (FEA) are compared with experimental results.

## 1. Introduction

The search for the improvement of materials, both innovative composites as well as whole structural systems, with special emphasis on aesthetics and environmental friendliness, has made prefabricated timber structures very popular. Wood exhibits multiple valuable properties: good strength to weight ratio, high thermal efficiency, simplicity in element shaping, relatively low cost—all these advantages make the wooden structures competitive as regards steel or reinforced-concrete. It still becomes more popular in the construction of multi-storey residential buildings utilizing large Cross Laminated Timber (CLT) panels.

In addition to requirements regarding the load-bearing capacity of the designed structure, serviceability requirements must also be taken into consideration. One of the crucial aspects of the problem of limiting the deflection according to building codes are the rheological phenomena related with viscoelastic properties of materials. In the case of CLT structures the global response of such a composite structure depends on individual viscoelastic properties of both wood and adhesive. These properties are influenced strongly by the relative humidity of the air, changes of temperature and biological corrosion. These factors were the subject of multiple elaborations [1,2]. Much research has already been done concerning the problem of the creep of the wood alone [3,4] and timber-concrete structures [5,6] as well as various strengthening of timber structures with the use of Fiber Reinforced Polymers (FRP) [7,8,9,10]. The problem of creep and relaxation of polymers and polymer matrices as well as the influence of temperature on the rheological response of material and modelling of such materials were investigated in [11,12,13]. An attempt to create a rheological model for the polyurethane adhesive has been made in [14]. The use of the generalized strain measures in constitutive modelling of nonlinear elastic adhesives was investigated in [15]. The possibilities of the improvement of plates with the use of innovative multilayer composite panels were investigated in [16].

Another field of research is the problem of the dynamic response of CLT structures, especially concerning their resistance to earthquakes [17,18,19,20]. In the opinion of the authors, connections using flexible polyurethane (FPU) adhesives are promising solutions for prefabricated timber structures, especially if the building is to be erected in a seismic area, where large energy dissipation is required. Broad research is still conducted in regards to the possibility of the application of FPUs, and first results have been published in [21,22].

The number of publications devoted to the experimental and numerical research and the current standardization of the time-dependent behavior of CLT slabs is relatively small, which emphasizes the need for extending the experimental and numerical research in this field. Additionally, the use of a thicker bond to increase the ductility of the component is a new solution and requires wide scope research to understand the behavior comprehensively.

As for the engineering design of CLT timber, Eurocode 5 (EC 5) [23] provides only a simplified design concept. In Annex B, we can find a method for the structural analysis of mechanically jointed beams, which is called the *γ*-method and was developed by Möhler [24]. The *γ*-method can be applied to CLT elements only with restrictions such as, for example, that the maximum number of layers is three. EC 5 does not specify a creep behavior for wood or calculation procedures for special cases. Final deflections *u_fin_* considering the effect of creep can be calculated depending on the duration of the load using appropriate formulas:

(1) $$u_{fin}=u_{inst,G}\cdot \left(1+k_{def}\right)+u_{instQ,1}\cdot \left(1+\psi_{2,1}\cdot k_{def}\right)+u_{instQ,i}\cdot \left(\psi_{0,i}+\psi_{2,i}\cdot k_{def}\right)$$

where *u_inst,G_*, *u_instQ,_*_1_ and *u_instQ,i_* represent the instantaneous deformation for permanent action *G*, the leading variable action *Q*_1_ and accompanying variable actions *Q_i_,* respectively. If the load is classified as permanent (more than 10 years), the final deflection should be calculated by increasing the instantaneous deformation by (1 + *k_def_*). The *k_def_* factor is the only coefficient reflecting the effect of creep and is dependent on the service class.

The aim of the presented research is to investigate further the problem of creep in CLT structures. In particular, our attention is focused on CLT beams which are made with the use of a relatively thick layer of highly deformable adhesive. This is contrary to the widespread practice of constructing the CLT structures with the adhesive layer of the smallest possible thickness. It is also a common approach to use relatively stiff epoxy adhesives. One should note, however, that in the joint with a thick (few millimeters) layer of flexible adhesive, the distribution of shear stress is more uniform than in the case of thin layers of stiff adhesives [25]. Two types of the CLT beams are examined, each one constructed with the use of different FPU, namely Sika^®^PS and Sike^®^PMM adhesives. All component materials—adhesives and wood—are also the subject of creep tests. Adhesives were tested in an uniaxial compression test with the use of cylinder specimens [26]; due to possible nonlinear characteristics of the material, the tests were performed for three levels of stress. The creep tests for timber were conducted with the use of two types of wooden planks of different thickness. Finally, a creep test was also performed for each type of the CLT beam.

Another problem investigated in this research is the analytical description and numerical prediction of viscoelastic behavior of the component materials as well as CLT beams. As a theoretical framework for material description, we were considering the two-element Generalized Maxwell Model, which is the simplest extension of the Standard Linear Solid model, for which still closed-form analytical solutions may be found. As regards numerical modelling, the Finite Element models of both CLT beam types were created with the use of the results of creep tests—then a viscoelastic analysis was carried out with the use of the Abaqus v. 6.14 software.

## 2. Materials and Methods

Viscous or rheological properties of materials may be investigated in several ways, primarily by conducting relaxation or creep tests, which enable finding the time-domain viscoelastic characteristics of the material, as well as by performing dynamic tests under oscillatory load, in which the extent of phase shift between stress and strain or magnitude of damping enable finding the frequency-domain characteristics. Creep tests—which are related with the most common rheological phenomenon observed in statically loaded structures—are among the simplest to be performed. Unlike the relaxation test in which continuous control of the loading factors is necessary, in the creep test one may use a constant gravitational load of fixed masses. In addition, the measurement of creep displacement may be performed directly with the use of simple dial sensors.

### 2.1. Spruce Wood

**Figure 1:**
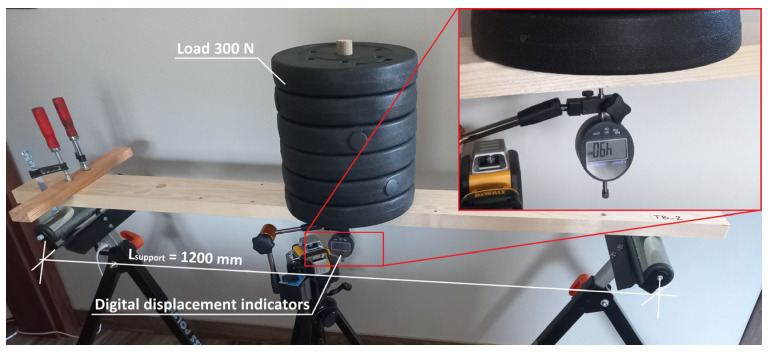
View of the test stand.

Creep tests were carried out for all component materials (spruce planks and polyurethane) as well as for the composite structure (CLT beams) itself. Spruce planks with two different cross-sections, type 1—100 × 22 mm—and type 2—118 × 30 mm—were subjected to three-point bending creep tests. Six samples were prepared for each type of cross-section marked from TB-1 to TB-6 (for type 1), and from TB-1A to TB-6A (for type 2). The test stand was prepared with the same dimensions as for a composite beam (CLT beams) described in Section 2.4. The length of the element was 1450 mm and the spacing between the supports was 1200 mm. A load of 300 N was applied in the middle of the span as shown in Figure 1. All samples were delivered in a single batch (after drying at the manufacturer). The moisture content of the wood was 12 ± 1.0% (mean value 11.4%). Moisture was measured using an electronic wood moisture meter HIT-2 (manufacturer Tanel from Gliwice, Poland) in three steps: post-delivery, pre-test and post-test.

The influence of time on the behavior of elements under constant load was considered. The viscoelastic properties needed to develop a numerical model of tested materials were determined. The displacement in the middle of the span was measured using digital indicators with a measuring range of 0–25.4 mm/L. The duration of the test was 7 days and deflection measurements were made with the following time schedule:

- after applying the load, instantaneous deflections,
- for the first 5 min, measurement every 1 min,
- for the first 60 min, measurement every 10 min,
- until the end of the test, measurement every 60 min.

The mean density of the lumber was 425 kg/m^3^. Creep testing was performed indoors, in uncontrolled conditions, the temperature of 22 ± 1.0 °C and a relative humidity (RH) of 42 ± 3%.

### 2.2. Adhesives

In this experimental study two-component polyurethane Sika^®^ PS and Sika^®^ PMM by Sika, Warsaw, Poland were used. Sika^®^ PMM is a solvent-free, flexible and high-quality recovery two-component polyurethane-based adhesive. In addition, connections using this material are additionally characterized by sound-absorbing properties and damping vibrations. Sika^®^ PS is a solvent-free, elastic, two-component, polyurethane-based adhesive. The basic properties of the adhesives are presented in the Table 1.

Both materials are part of an innovative system for bonding structural and non-structural wood and wood-based materials developed at the Cracow University of Technology. Adhesives are available in Poland on special order and are manufactured by Sika Poland. For the preparation of small samples for the compression test as well as for the bonding of CLT beams, the adhesives were mixed in a weight ratio (component A:component B) of 100:10 and 100:12 for Sika^®^ PS and Sika^®^ PMM respectively. The mixing procedure was according to technical data sheets. Component A is a base (polyurethane resin) and component B is a hardener. More details, such as the aspect of durability of Sika^®^PS polyurethane, can be found in [27].

### 2.3. Creep Test for Adhesives

**Figure 2:**
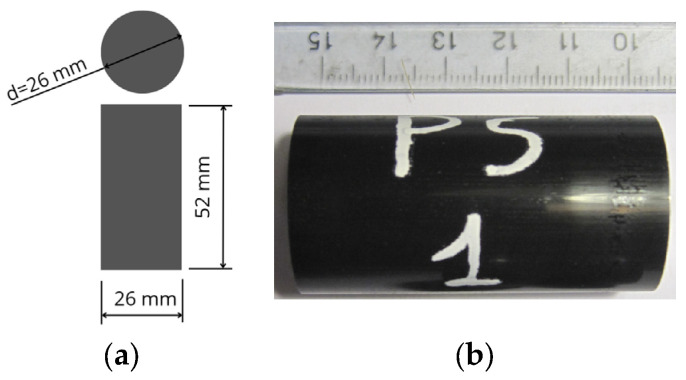
Sample subjected to uniaxial compression creep tests: (**a**) sample dimensions; (**b**) view of the sample.

Uniaxial compression creep tests were performed for two flexible polyurethane adhesives: Sika^®^ PS and Sika^®^ PMM. The samples were prepared in accordance with ASTM D 695-02 [28] in the form of cylinders whose length is twice its diameter. However, the dimensions were changed from 12.7 × 25.4 mm to 26 × 52 mm due to the dimensions of the molds used to prepare samples. All samples were prepared 28 days before the start of the test and conditioned in a room at stable temperature of 22 ± 2.0 °C and humidity of 50 ± 5%. The view of the sample is shown in Figure 2.

The test consisted of three load steps and the number of samples for each load level was three. The levels of load were determined separately for polyurethane Sika^®^ PS and Sika^®^ PMM. The third load step was set as the stress value corresponding to the strain equal to 1/3 of the maximum strain for the linear range in the compression test. The other steps were determined dividing by three the segment found. Next, the value of the load that should be applied to the samples depending on the considered step was determined. The parameters are presented in Table 2.

**Figure 3:**
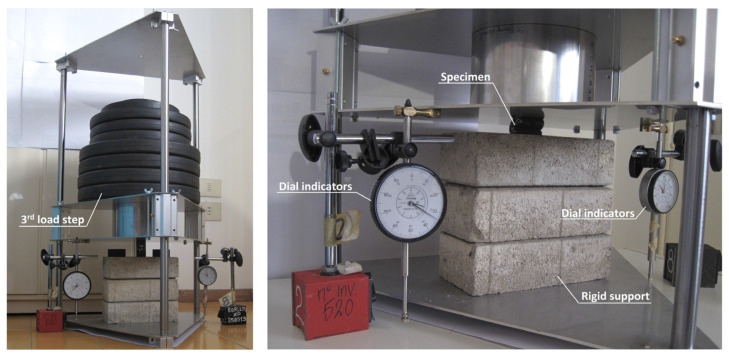
View of the test stand.

For each step, the sample was loaded for not less than 100 h and measurements were made in the following scheme: 1, 6, 12, and 30 min; 1, 2, 5, 20, 50 and 100 h. Two dial indicators with an accuracy equal to 0.01 mm were used for the measurements of deformation as shown in Figure 3. The final deformation was presented as the average value of two measurements. The tests were conducted at a temperature of 22 ± 2 °C, and a relative humidity of 50 ± 5%.

### 2.4. CLT Beams

**Figure 4:**
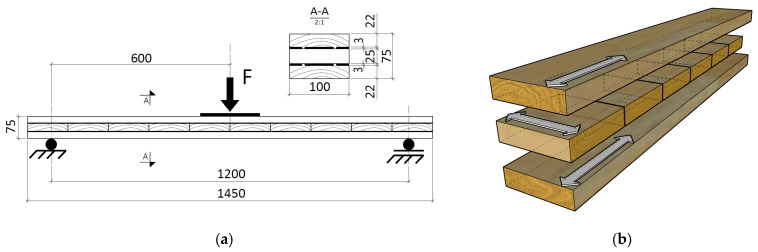
Creep test for CLT beams: (**a**) schematic view; (**b**) the scheme illustrating the construction of CLT beams.

Three-point bending creep tests were performed for CLT beams. The configuration and geometry of the test specimens are illustrated in Figure 4. One beam glued with polyurethane Sika^®^ PS and Sika^®^ PMM was prepared. Each sample contained external wooden lamella in a longitudinal arrangement (0°) and a middle lamella in a perpendicular arrangement (90°), see Figure 4b. External wooden lamella with an initial height of 25 mm were reduced to a height of 22 mm (cross-section as in the wood creep tests for spruce planks type 1), leaving technological guide bars thanks to which a precise thickness of the adhesive layer of 3 mm was obtained. The lamellas were glued plane-to-plane, without finger joints.

**Figure 5:**
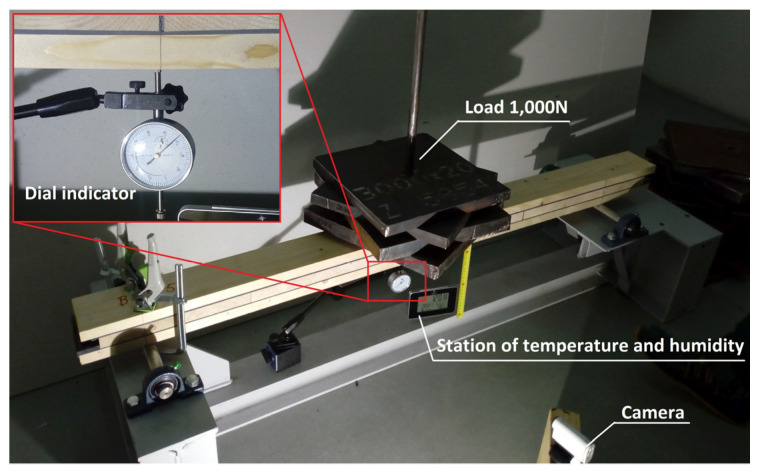
View of the test stand for CLT beam.

Beams with a cross-section of 100 × 75 mm and a total length of 1450 mm were tested. The spacing of supports was 1200 mm. The load of 1000 N in the form of steel plates was applied in the middle of the span as shown in Figure 5. The dial indicator with an accuracy equal to 0.01 mm was used for the measurements of displacement. The beams were prepared 21 days before the start of the tests and seasoned in a room with a constant temperature of 20 ± 2 °C and a relative humidity 50 ± 5%. During the test, the moisture content of the CLT beams was 12 ± 1.0%.

For the CLT beam made with the polyurethane Sika^®^ PS the load was applied for 72 h, and deflection measurements were carried out in the following schedule: 0, 1, 2, 4, 8, 12, 16, 24, 48 and 72 h. In the case of CLT beam made with the polyurethane Sika^®^ PMM the load was applied for 24 h, and the deflection measurement was carried out as follows: 0, 1, 2, 4, 8, 12 and 24 h.

### 2.5. Theoretical Description

The most commonly used approach in an analytical description of viscoelastic behavior of materials is the use of the theory of linear viscoelastic Generalized Maxwell Model (which is also termed the Maxwell–Wiechert Model [29,30]).

**Figure 6:**
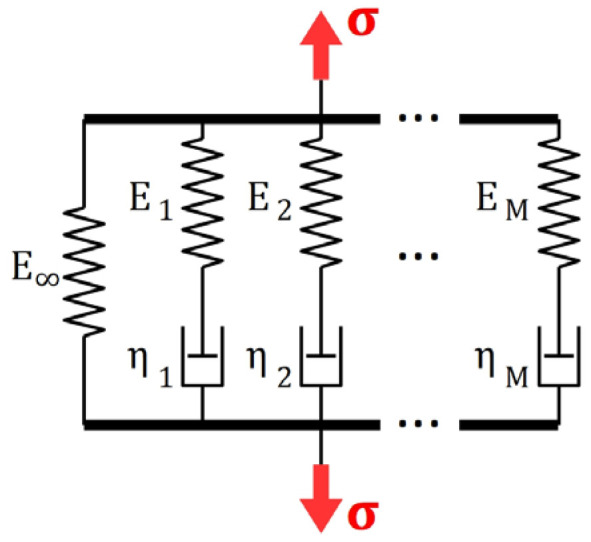
The M-element Generalized Maxwell Model.

The relaxation function corresponding with any M-element GMM (Figure 6) characterized by stiffness moduli $E_{\infty },\;E_{1},\ldots ,E_{M}$ and viscosities $\eta_{1},\ldots ,\eta_{M}$ may be easily found as a solution a linear ordinary differential equation, since roots of a characteristic polynomial

(2) $$1+\left(\tau_{1}+\tau_{2}+\ldots +\tau_{M}\right)s+\ldots +\left(\tau_{1}\cdot \tau_{2}\cdot \ldots \cdot \tau_{M}\right)s^{M}=0$$

related with that equation are simply the relaxation times $\tau_{i}=\frac{\eta_{i}}{E_{i}}$ (i=1,…,M). It may be easily verified with the use of Vieta’s formulas. Constants of integration are found according to the following initial conditions:

(3) $$\sigma \left(0\right)=E_{0}\varepsilon_{0},\;\;{\left.\frac{d^{m}\sigma }{dt^{m}}\right|}_{t=0}=0\;\left(m=1,\ldots ,M-1\right)$$

where $E_{0}=E_{\infty }+E_{1}+E_{2}+\ldots E_{M}$ is the instantaneous Young’s modulus. The constants of integration may be determined with the use of properties of the Vandermonde matrices. Then the relaxation function $g_{R}\left(t\right)$ defining the hereditary constitutive relation

(4) $$\sigma \left(t\right)=G_{0}\int_{0}^{t}g_{R}\left(t-\tau \right)\frac{d\varepsilon \left(\tau \right)}{d\tau }\;d\tau$$

may be expressed with the use of the following expression:

(5) $$g_{R}\left(t\right)=1-\overset{M}{\underset{i=1}{\sum }}g_{i}\left(1-e^{-\frac{t}{\tau_{i}}}\right)\;\mathrm{where}\;\;\;g_{i}=\left(1-\frac{E_{\infty }}{E_{0}}\right)\frac{\tau_{i}^{n-1}}{\prod_{\left\{\begin{array}{l} j=1,\ldots ,M\\ j\neq i \end{array}\right.}\left(\tau_{j}-\tau_{i}\right)}$$

An explicit closed-form formula for the relaxation function may be found for any number M of Maxwell elements in GMM—knowing this expression, it is possible to find the parameters of the GMM with the use of curve fitting algorithms. However, it is not the case as regards the creep function because the characteristic polynomial corresponding with the ODE describing the creep test is a polynomial of degree M, the roots of which cannot be found easily. According to the Ruffini–Abel theorem, it may be done in general case only for M≤4, however, already for M=3, the formulas become intractable.

An alternative approach in determining the creep function is the use of the correspondence principle [31], the application of which has been extended for heterogeneous and composite bodies [32,33]. If the boundary conditions are time-independent, then if the system of governing equations of the boundary value problem (BVP) of theory of viscoelasticity are transformed under the Laplace integral transform, then a corresponding BVP of theory of elasticity is obtained. Then, if relaxation function $g_{R}$ is known, the normalized creep function $j_{C}$ may be found according to the following relation:

(6) $$j_{C}\left(t\right)=L^{-1}\left[-\frac{L\left[K_{R}\right]}{1+L\left[K_{R}\right]}\right]\;\mathrm{where}\;\;\;K_{R}=\frac{\partial g_{R}}{\partial t}$$

While the above relation may emerge efficient in simple cases, finding the inverse Laplace transform for large M cannot be done analytically and numerical computation may suffer from singularities which are found in Laplace transforms of exponential functions present in Equation (5). The issues mentioned above make it difficult to analytically describe the viscoelastic properties of the considered material basing only on creep tests, which are still the simplest rheological tests to be carried out.

In the Section 4, a closed-form explicit formula for creep function is derived for a two-element GMM (M=2). Young’s moduli of the spring elements as well as the retardation times are then determined for the considered theoretical model according to the experimental results.

### 2.6. Numerical Analysis

The data from the creep tests performed for wood and adhesives were used in the Abaqus software [34] in order to characterize the viscoelastic properties of tested materials. A FE element model of the CLT beam was elaborated and linear viscoelastic analysis of creep of such a model was carried out. Obtained results were compared with the recorded creep deformation of CLT beams.

## 3. Results of Creep Tests

**Figure 7:**
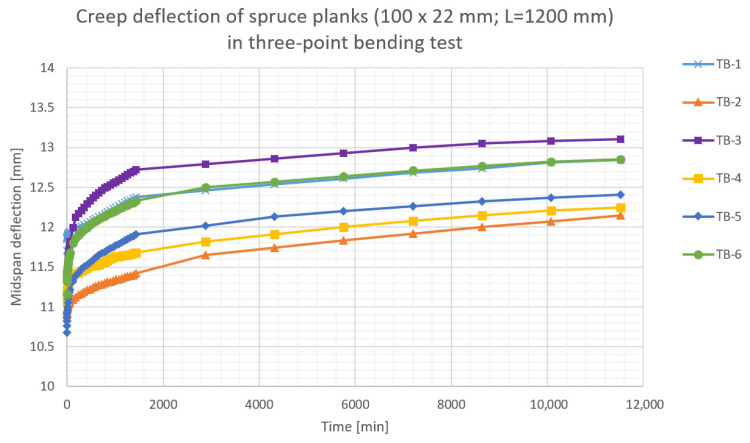
Creep deflection of spruce planks (100 × 22 mm) in three-point bending.

**Figure 8:**
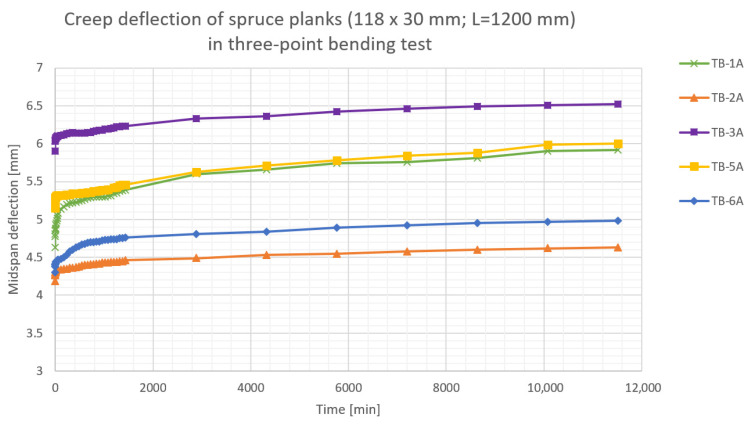
Creep deflection of spruce planks (118 × 30 mm) in three-point bending.

**Figure 9:**
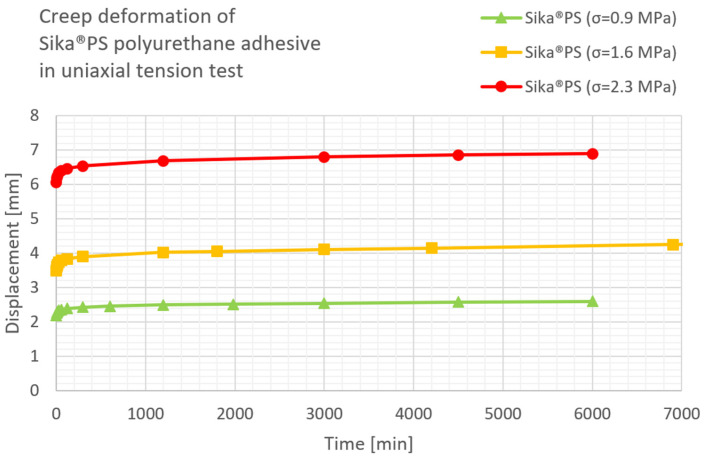
Creep deformation of Sika^®^PS adhesive in uniaxial compression test under three load levels.

**Figure 10:**
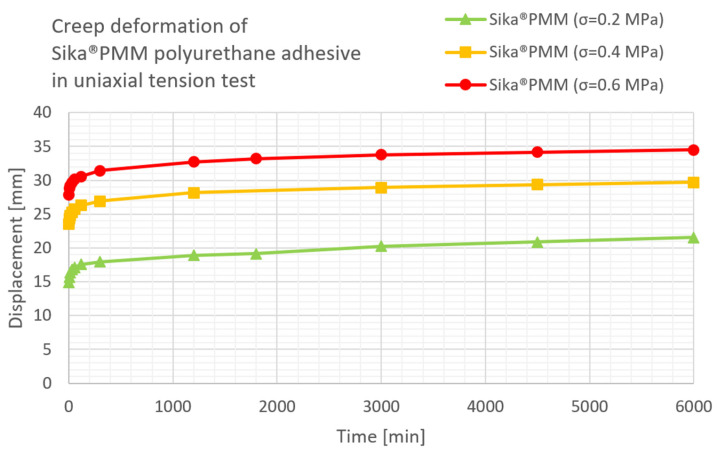
Creep deformation of Sika^®^PMM adhesive in uniaxial tension test under three load levels.

**Figure 11:**
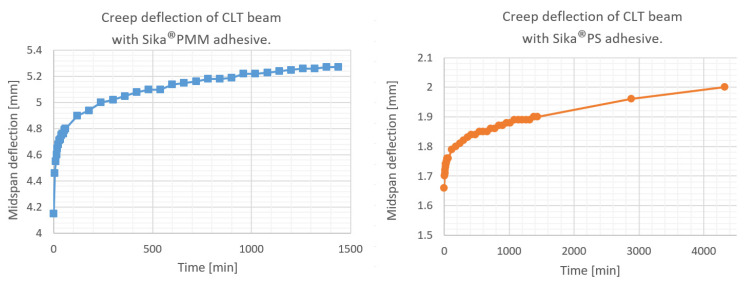
Creep deflection of CLT beams in three-point bending.

The results of the performed creep tests are presented in Figure 7, Figure 8, Figure 9, Figure 10 and Figure 11. Creep deflection of wooden planks in three-point bending is presented in Figure 7 (plank’s cross-section: 100 × 22 mm) and in Figure 8 (plank’s cross-section: 118 × 30 mm). Temperature, relative humidity (RH), instantaneous deflections (*u_inst_*) and deflections after each 24 h during the first week of deformation are given in Table 3 for the planks 22 mm thick and in Table 4 for planks 30 mm thick.

The results of the performed tests indicate, that the wood used for construction of CLT beams corresponded roughly with C24 class, according to EN 338:2016 [35].

The results of the uniaxial compression test for three load levels applied to polyurethane Sika^®^ PS and Sika^®^ PMM, according to [26], are presented in Figure 9 and Figure 10, respectively.

Creep deflection of CLT beams in three-point bending is presented in Figure 11. Temperature, relative humidity, instantaneous deflections of the CLT beam with polyurethane Sika^®^ PS as well as deflections during the first day and after 2 and 3 days are given in Table 5. In Table 6 the data are presented for the CLT beam with PMM polyurethane—deflections are given only for the first 24 h.

## 4. Description of Viscoelastic Properties of the Materials with the Use of the Generalized Maxwell Model

**Figure 12:**
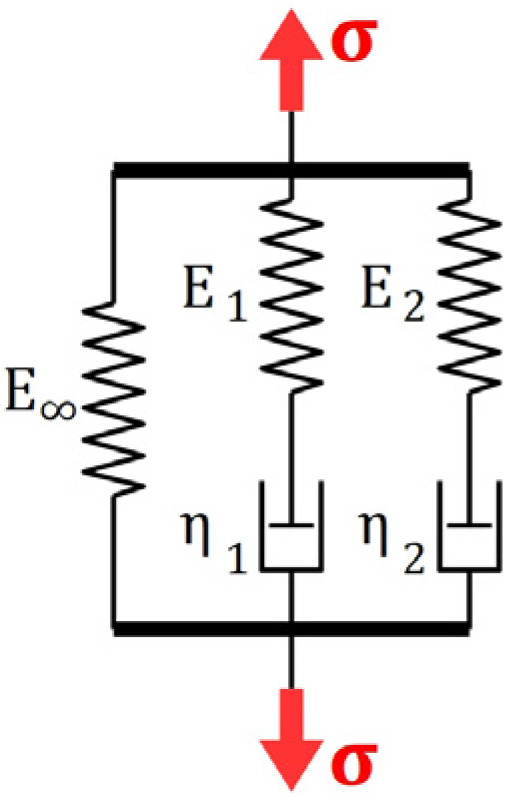
The 2-element Generalized Maxwell Model.

The two-element Generalized Maxwell model is considered (Figure 12).

The local (differential) form of the constitutive relation is as follows:

(7) $$\sigma +\left(\tau_{1}+\tau_{2}\right)\dot{\sigma }+\tau_{1}\tau_{2}\ddot{\sigma }=E_{\infty }\varepsilon +\left[\left(E_{\infty }+E_{1}\right)\tau_{1}+\left(E_{\infty }+E_{2}\right)\tau_{2}\;\right]\dot{\varepsilon }+E_{0}\tau_{1}\tau_{2}\ddot{\varepsilon }$$

where instantaneous Young’s modulus is equal $E_{0}=\left(E_{\infty }+E_{1}+E_{2}\right)$. In the case of the creep test, we have $\sigma \left(t\right)=\sigma_{0}=const.$, which yield the following ODE:

(8) $$E_{\infty }\varepsilon +\left[\left(E_{\infty }+E_{1}\right)\tau_{1}+\left(E_{\infty }+E_{2}\right)\tau_{2}\;\right]\dot{\varepsilon }+E_{0}\tau_{1}\tau_{2}\ddot{\varepsilon }=\sigma_{0}$$

and corresponding initial conditions:

(9) $$\left\{\begin{array}{l} \varepsilon \left(t=0\right)=\frac{\sigma_{0}}{E_{0}}\\ \dot{\varepsilon }\left(t=0\right)=0 \end{array}\right.$$

The solution of the above initial value problem (IVP) is given by the following formula:

(10) $$\varepsilon \left(t\right)=\frac{\sigma_{0}}{E_{0}}j_{C}\left(t\right)\;\;\;\;\;\mathrm{where}\;\;\;\;\;j_{C}\left(t\right)=1+\overset{2}{\underset{i=1}{\sum }}c_{i}\left(1-e^{-\frac{t}{T_{i}}}\right)$$

where creep moduli are equal to

(11) $$c_{1}=\left(\frac{E_{0}}{E_{\infty }}-1\right)\frac{T_{1}}{\left(T_{1}-T_{2}\right)},\;\;c_{2}=\left(\frac{E_{0}}{E_{\infty }}-1\right)\frac{T_{2}}{\left(T_{2}-T_{1}\right)}$$

and corresponding retardation times are equal to

(12) $$\begin{array}{c} T_{1}=\frac{2E_{0}\tau_{1}\tau_{2}}{\left(E_{\infty }+E_{1}\right)\tau_{1}+\left(E_{\infty }+E_{2}\right)\tau_{2}+\sqrt{{\left[\left(E_{\infty }+E_{1}\right)\tau_{1}+\left(E_{\infty }+E_{2}\right)\tau_{2}\right]}^{2}-4E_{0}E_{\infty }\tau_{1}\tau_{2}}}\\ T_{2}=\frac{2E_{0}\tau_{1}\tau_{2}}{\left(E_{\infty }+E_{1}\right)\tau_{1}+\left(E_{\infty }+E_{2}\right)\tau_{2}-\sqrt{{\left[\left(E_{\infty }+E_{1}\right)\tau_{1}+\left(E_{\infty }+E_{2}\right)\tau_{2}\right]}^{2}-4E_{0}E_{\infty }\tau_{1}\tau_{2}}} \end{array}$$

The ratio $\frac{E_{0}}{E_{\infty }}$ as well as the retardation times $T_{1}$ and $T_{2}$ were found for the spruce wood, PS and PMM polyurethanes according to the obtained experimental results. The creep deformation was sampled with greater frequency at the beginning of the creep process, which may result in poor fitting of the theoretical curve to measurements recorded later. In order to avoid such a situation the Synthetic Minority Over-Sampling Technique (SMOTE) was used [36]. Synthetic data points were generated randomly within each region $\left(t_{i};t_{i+1}\right)\times \left(\varepsilon_{i};\varepsilon_{i+1}\;\right)$ and their number was related with the smallest distance between opposite corners this region. Optimal model parameters were found with the use of the direct search method for each material and for each load level applied to polymers. The results are presented in Table 7.

Parameter $T_{2}$ is not listed in the Table 1, because an optimal value was always found as close to zero. This issue is discussed in the Discussion Section.

It is worth noting that the GMM may also be used for an approximate description of the whole viscoelastic system, i.e., the CLT beam. The stiffness of spring elements in the GMM is then interpreted in terms of global flexural rigidity,

(13) $$k=\frac{48\;EI}{L^{3}}$$

of such a CLT beam. Table 8 presents the parameters of the GMM applied for the description of CLT beams.

## 5. Finite Element Model and Results of Numerical Analysis

The Finite Element model was created for both types of the CLT beams. Time-domain viscoelastic analysis was performed with the use of the Abaqus software. All materials were considered linear viscoelastic materials. Mechanical properties of the component materials were assumed according to the results of performed experiments. Instantaneous Young’s moduli were measured directly. Wooden panels were modelled as isotropic solids, which is justified by the uniaxial character of the stress and strain state in bending. The Young’s modulus parallel to grain $E_{0}$ for the top and bottom wooden panels were assumed according to the instantaneous flexural rigidity recorded in the three-point bending tests performed on wooden planks. Since considerable variation of experimental results was observed as regards determination of mechanical properties of component materials, multiple FEA were carried out in order to adjust the material parameters of the FE model properly. Young’s modulus and normalized creep function for spruce wood was assumed as an arithmetic mean of the quantities determined for the wooden specimens 22 mm thick. Poisson’s ratio was assumed to be equal to 0.36 according to EN 338 [35]. The Young’s modulus perpendicular to grain for the middle wooden panels was assumed to be equal to $E_{90}\approx 0.033E_{0}$, according to [35]. Instantaneous Young’s modulus of the Sika^®^PS adhesive was assumed to be an arithmetic mean of the moduli corresponding with different strain rates. The value of the Young’s modulus of the Sika^®^PMM adhesive had to be adjusted, since true stress in the adhesive layer in CLT beam was much greater than the one assumed in creep tests. Finding the initial tangent Young’s modulus for such small stresses may also be flawed for such a greatly extensible polymer. For these reasons the Young’s modulus of PMM adhesive was adjusted according to [21].

Long-term stiffness moduli were estimated according to the slope of the creep curve. Creep test data were used for determination of the viscoelastic constitutive relation making use of the Prony series representation, involving up to 13 terms. The maximum allowable average RMS error was 2%. The creep function for middle wooden panels loaded perpendicularly to grain was assumed to be five times greater than the one corresponding to bending (tension/compression) parallel to grain [37]. Creep functions for adhesives were determined as an average of functions corresponding with different strain rates. For all materials normalized creep functions were assumed the same for both shear and bulk deformation.

**Figure 13:**
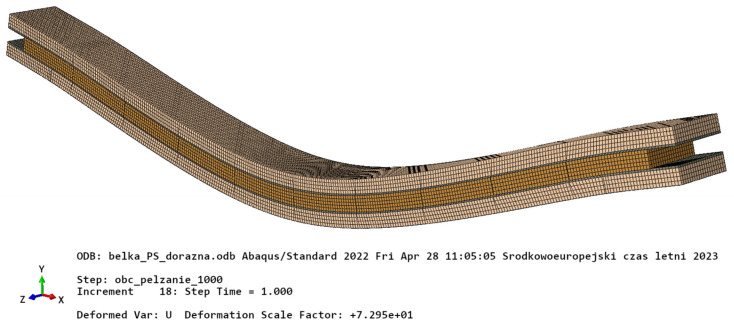
The FE model of the CLT beam. Deformed configuration.

Kinematic boundary conditions were modelled as fixed vertical displacement constraints within support areas 2 mm wide. The external load was modelled as uniformly distributed pressure applied at the top of the beam on the area the size of which corresponded with the one used in the experiments. Fine regular orthogonal mesh was assumed due to small dimensions of the adhesive layers and support areas. The total number of ca. 87,000 standard linear hexahedral finite elements with ca. 295,000 degrees of freedom were used (Figure 13).

**Figure 14:**
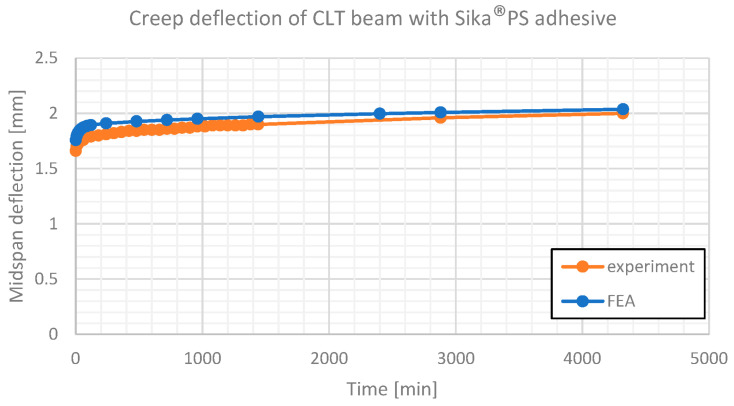
Creep deflection of CLT beam with Sika^®^PS adhesive. Comparison of experimental results and numerical estimates.

**Figure 15:**
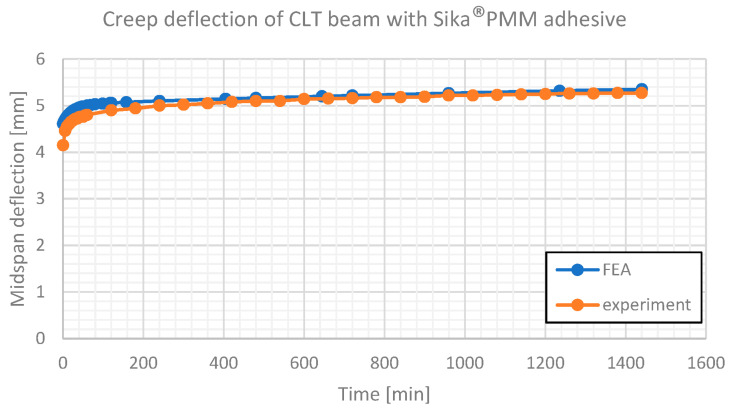
Creep deflection of CLT beam with Sika^®^PMM adhesive. Comparison of experimental results and numerical estimates.

The creep deformation of two considered beams is presented in Figure 14 and Figure 15.

## 6. Discussion

Analysis of the results of material testing indicates that both in the case of wood as well as in the case of polymers, material characteristics vary to a considerable extent, despite the fact that all experiments were carried out in approximately constant humidity and temperature conditions. Observed small variations of relative humidity and temperature were assumed to have minor impact on the response of the beams and therefore they were not taken into consideration. In the case of wood this could be at least partially explained by small differences in the moisture content. It has also been found that the thicker planks were most probably of slightly different grade, as they exhibited a noticeably smaller stiffness and larger creep.

It has been marked that parameter $T_{2}$ in the two-element GMM model was always found to be approximately 0 for all considered materials. This is due to the fact that the lowest of the coefficients $c_{1}$ and $c_{2}$ is necessarily negative. Values of $T_{1}$ and $T_{2}$ are considered positive, since negative retardation times would result in exponential growth of creep, which is against experimental evidence. For positive retardation time $T_{2}$ and negative coefficient $c_{2}$ one obtains monotonically decreasing component of the creep function, which is also inconsistent with physical nature of creep. As a result, the optimal parameters, which fit the theoretical curve to the experimental results, are such that the term with the negative coefficient is cancelled, which requires the corresponding retardation time to be equal to 0.

It can be observed that the CLT beams exhibit slightly larger creep than the one estimated by the Finite Element Analysis. Among the most probable reasons, one should name insufficient accuracy of determination of material parameters, which is due to relatively large variation of the results of material testing. Another explanation could be related to the fact that for each material both creep functions—shear and bulk functions—were assumed the same, according to the uniaxial test for polymers and bending test for wood. One may suspect that in fact retardation times for shear deformation are different than for bulk deformation. This problem may be of greater importance regarding the adhesives, since adhesive layers are primarily sheared in CLT beams—in such a situation creep function determined in a uniaxial test (which is in fact a combined bulk + shear deformation state) may emerge to be inappropriate.

## 7. Conclusions

The performed analysis enables formulation of the following conclusions:

- The creep rates of timber and the considered polyurethane adhesive are of a similar order of magnitude. Creep parameter $\phi =\left(\frac{E_{0}}{E_{\infty }}-1\right)$ was found between 10% and 20% for most wooden samples as well as for Sika^®^PS adhesive. The Sika^®^PMM adhesive exhibits somewhat larger creep.
- Linear theory of viscoelasticity provides estimates of creep deformation which are in fair agreement with the experimental results. This means that non-linear constitutive characteristics of adhesives are of minor importance unless the strain is sufficiently small.
- The two-element Generalized Maxwell Model cannot be fitted to the creep data in any better way than one-element GMM due to the fact that the specific mathematical form of the general solution of the governing ODE requires one of the creep moduli to be negative (which is against the nature of the creep phenomenon) or equal to 0.
- In order to obtain better accuracy in predicting the creep deformation of CLT beams it is required to examine the shear creep function and bulk deformation creep function independently.

## Figures and Tables

**Table 1:** Properties of adhesives.

Adhesive Types	Young Modulus (MPa)	Ultimate Elongation (%)	Tensile Strength (MPa)	Density (g/cm^3^)
PS	20	30	2.6	1.40
PMM	3	100	1.1	0.95

**Table 2:** Load steps converted in weight [kg] for each material.

Material	Load					
	Initial Load		2nd Step		3rd Step	
	[MPa]	[kg]	[MPa]	[kg]	[MPa]	[kg]
PS	0.9	48.71	1.6	86.6	2.3	124.48
PMM	0.2	10.82	0.4	21.65	0.6	32.47

**Table 3:** Creep deflection of spruce planks (100 × 22 mm) in three-point bending during first 7 days.

Specimen		Time [Days]							
		$0\;(u_{inst})$	1	2	3	4	5	6	7
TB-1	u [mm]	11.70	12.38	12.46	12.54	12.61	12.68	12.74	12.81
TB-2	u [mm]	10.86	11.42	11.65	11.74	11.83	11.92	12.00	12.07
TB-3	u [mm]	11.13	12.72	12.79	13.86	12.93	13.00	13.05	13.08
TB-4	u [mm]	11.07	11.68	11.82	11.91	12.00	12.08	12.15	12.21
TB-5	u [mm]	10.68	11.91	12.02	12.13	12.20	12.26	12.32	12.37
TB-6	u [mm]	11.16	12.33	12.50	12.57	12.64	12.71	12.77	12.82
	temp. [°C]	22.3	22.2	22.3	22.0	22.0	22.0	22.2	22.0
	RH [%]	41	42	41	44	45	42	42	41

**Table 4:** Creep deflection of spruce planks (118 × 30 mm) in three-point bending during first 7 days.

Specimen		Time [Days]							
		$0\;(u_{inst})$	1	2	3	4	5	6	7
TB-1A	u [mm]	4.63	5.39	5.60	5.66	5.74	5.76	5.81	5.90
TB-2A	u [mm]	4.19	4.46	4.49	4.53	4.55	4.58	4.60	4.62
TB-3A	u [mm]	5.90	6.23	6.33	6.36	6.42	6.46	6.49	6.51
TB-5A	u [mm]	5.15	5.46	5.63	5.71	5.78	5.84	5.88	5.99
TB-6A	u [mm]	4.30	4.76	4.81	4.84	4.89	4.92	4.95	4.97
	temp. [°C]	22.3	22.2	22.3	22.0	22.0	22.0	22.2	22.0
	RH [%]	41	42	41	44	45	42	42	41

**Table 5:** Creep deflection of the CLT beam with Sika^®^PS adhesive in three-point bending during first 3 days of creep.

Adhesive Type		Time [Hours]									
		0	1	2	4	8	12	16	24	48	72
Sika^®^PS	u [mm]	1.66	1.76	1.79	1.81	1.84	1.86	1.88	1.90	1.96	2.00
	temp. [°C]	17.7	17.5	17.6	17.8	18.1	18.2	18.2	18.5	19.2	19.7
	RH [%]	61	62	63	63	61	61	59	58	58	60

**Table 6:** Creep deflection of the CLT beam with Sika^®^PMM adhesive in three-point bending during the first day of creep.

Adhesive Type		Time [Hours]							
		0	1	2	4	8	12	16	24
Sika^®^PMM	u [mm]	4.15	4.80	4.90	5.00	5.10	5.16	5.22	5.27
	temp. [°C]	17.2	17.2	17.2	17.2	17.4	17.5	17.5	17.5
	RH [%]	35	36	36	36	36	36	37	36

**Table 7:** Parameters of the 2-element Generalized Maxwell Model used for analytical description of viscoelastic properties of the examined materials.

Material	$E_{0}$ [MPa]	$E_{\infty }$ [MPa]	$T_{1}$ [s]	$\frac{E_{0}}{E_{\infty }}-1$
Spruce wood (100 × 22)	10,965	9776.2	74,327	12.1%
Spruce wood (118 × 30)	8415.0	7334.6	112,150	14.7%
Sika^®^ PS (0.9 MPa)	21.020	18.014	28,546	16.7%
Sika^®^ PS (1.6 MPa)	23.296	18.378	186,710	26.8%
Sika^®^ PS (2.3 MPa)	19.189	17.027	30,660	12.7%
Sika^®^ PMM (0.2 MPa)	0.692	0.494	64,433	40.1%
Sika^®^ PMM (0.4 MPa)	0.877	0.708	33,432	23.9%
Sika^®^ PMM (0.6 MPa)	1.116	0.891	69,492	25.3%

**Table 8:** Parameters of the 2-element Generalized Maxwell Model used for analytical description of viscoelastic properties of the examined CLT beams.

Element	$k_{0}$ [kN/m]	$k_{\infty }$ [kN/m]	$T_{1}$ [s]	$\frac{k_{0}}{k_{\infty }}-1$
CLT beam (PS)	602.41	509.01	41,937	18.3%
CLT beam (PMM)	240.96	193.11	5751.5	24.8%

## Data Availability

Restrictions apply to the availability of these data. Data was obtained from PalettenWerk Kozik Sp. z o. o and may be individually shared at reasonable request with the permission of PalettenWerk Kozik Sp. z o. o.
